# Anti-herpes virus activities of bioactive fraction and isolated pure constituent of *Mallotus peltatus*: an ethnomedicine from Andaman Islands

**DOI:** 10.1186/1743-422X-9-98

**Published:** 2012-05-24

**Authors:** Paromita Bag, Debprasad Chattopadhyay, Hemanta Mukherjee, Durbadal Ojha, Nilanjan Mandal, Mamta Chawla Sarkar, Tapan Chatterjee, Gobardhan Das, Sekhar Chakraborti

**Affiliations:** 1 ICMR Virus Unit, ID & BG Hospital, General Block 4, First floor, 57 Dr Suresh Chandra Banerjee Road, Beliaghata, Kolkata, 700010, India; 2 Division of Virology, National Institute of Cholera & Enteric Diseases, Kolkata, India; 3 Department of Pharmaceutical Technology, Jadavpur University, Kolkata, India; 4 Immunology Group, International Centre for Genetic Engineering and Biotechnology, New Delhi, India

**Keywords:** *Mallotus peltatus*, Ethnomedicine, Antiviral activity, Herpes simplex virus, Ursolic acid

## Abstract

**Background:**

Viral infections, particularly the infections caused by herpes simplex virus (HSV), represent one of the most serious public health concerns globally because of their devastating impact. The aim of this study was to evaluate the antiviral potential of methanolic crude extract of an ethnomedicine *Mallotus peltatus*, its active fraction and pure compound, against HSV-1 F and HSV-2 G.

**Result:**

The cytotoxicity (CC_50_, the concentration of 50% cellular toxicity), antiviral effective concentration (EC_50_, the concentration required to achieve 50% protection against virus-induced cytopathic effect), plaque reduction and the selectivity index (SI, the ratio of CC_50_ and EC_50_) was determined. Results showed that the crude methanolic extract of *M. peltatus* possessed weak anti-HSV activity. In contrast, the active fraction A and isolated ursolic acid from fraction A exhibited potent antiherpesvirus activity against both HSV-1 (EC_50_ = 7.8 and 5.5 μg/ml; SI = 22.3 and 20) and HSV-2 (EC_50_ = 8.2 and 5.8 μg/ml, and SI = 21.2 and 18.97). The fraction A and isolated ursolic acid (10 μg/ml) inhibited plaque formation of HSV-1 and HSV-2 at more than 80% levels, with a dose dependent antiviral activity, compared to acyclovir. The time response study revealed that the anti-HSV activity of fraction A and isolated ursolic acid is highest at 2–5 h post-infection. Moreover, the time kinetics study by indirect immunofluorescence assay showed a characteristic pattern of small foci of single fluorescent cells in fraction A- treated virus infected cells at 2 h and 4 h post-infection, suggesting drug inhibited viral dissemination. Further, the PCR study with infected cell cultures treated with fraction A and isolated ursolic acid at various time intervals, failed to show amplification at 48–72 h, like acyclovir treated HSV-infected cells. Moreover, fraction A or isolated ursolic acid showed no interaction in combination with acyclovir.

**Conclusion:**

This study revealed that bioactive fraction A and isolated ursolic acid of *M. peltatus* has good anti-HSV activity, probably by inhibiting the early stage of multiplication (post-infection of 0–5 h), with SI value of 20, suggesting its potential use as anti-HSV agents.

## Introduction

Herpes simplex viruses (HSV) are a common human pathogen that causes *herpes labilesherpes genitalis*, keratitis and encephalitis. The HSV infection caused by type-1 (HSV-1) and type-2 (HSV-2) is mainly transmitted by close personal contact, and the virus can establishes lifelong latent infection in sensory neurons with recurrent lesions [1]. *Herpes genitalis*, usually caused by HSV-2, spread silently through sex, wreaks enormous financial and emotional damage due to its silent epidemic potential, and can cause life threatening infection in immunocompromised people and neonates [2]. Moreover, HSV-2 is a high risk factor for acquisition of HIV infection [3,4] and there is a synergistic relationship between HIV and HSV [5-7]. A recent study showed that HSV-suppressive therapy greatly reduced genital and plasma HIV-1 RNA levels in co-infected patients [8]. Hence, the risk of acquiring or transmitting HIV infection can be greatly decreased by reducing the spread of genital herpes.

Extensive and long term clinical use of anti-herpesvirus agents like acyclovir, and its derivatives ganciclovir, foscarnet results severe side effects and drug-resistant viruses [9-11]. Further, acyclovir is reported to incorporate into the cellular DNA, yielding adverse drug reactions and thus, unsuitable for pregnant women [12] and neonates [13,14]. Moreover, the major determinants of effective immunity against HSV infection is not yet identified [15], and animal efficacy has not predicted success in humans [16]. Furthermore, the therapeutic vaccines failed to induce antibody-specific responses to protect recipients from recurrences [15]. Therefore, there is an unmated and urgent need for cheap, readily available, less toxic alternate agents to control and prevent HSV infection and its transmission. Ethnomedicinal plants offer a potential alternative because of their wide use in folklore medicine and some have promising therapeutic potential [17].

One of the widely used folklore medicine *Mallotus peltatus* (Geist) Muell. Arg. (Euphorbiaceae), known as Pataque and Obottacke by Onge and Kamala by local people, is a panatropical shrub endemic to the inland forests of Chidiyatappu, Baratang, Jarawa Creek, and Interview Islands of Andamans. The decoction of *M. peltatus* leaves is widely used among the tribal populations of Bay Islands, India, to treat skin and intestinal ailments [18], and stomachache [19]. However, till date there is no scientific validation of the use of this ethnomedicine. As our ongoing effort to identify potential therapeutic lead from ethnomedicinal source we have evaluated several ethnomedicines including *M. peltatus* for antimicrobial [20], antiinflammatory and related activities [20-22]. Based on traditional use in skin infections the aim of the present work is to evaluate, for the first time, the *in vitro* antiviral activity of the crude methanolic extract, most active fraction, and the isolated compound(s) from the active fraction of *M. peltatus* leaf.

## Materials and methods

### Plant materials

The leaves of *M. peltatus* (Geist.) Muell. Arg. was collected from the rain forests of Middle and Southern Andaman (Chidiyatappu, Baratang and Jaroaw Creek), India, throughout the year. The voucher specimens were identified by Dr. Sreekumar, Senior Scientist, Botanical Survey of India (BSI), and deposited in the Herbarium Section (Herbarium No. 9221) of the BSI, Andaman and Nicobar Circle, Port Blair, for future reference. The leaves were separately dried in shade, pulverized by a mechanical grinder and passed through 40-mesh sieve to get the fine powder.

### Preparation of extracts

Coarsely powdered dry leaves (980 g) were extracted with 95% methanol for 72 h at room temperature [23]. The whole extract was collected, filtered, and solvent evaporated to dryness under reduced pressure in a Eyela Rotary Evaporator (Japan) at 40–45°C. The concentrated extract was aliquoted in amber-coloured bottles and kept in dessicator for further use. The w/w yield of the prepared extract was 8.7 ± 0.21.

### Phytochemical screening and chemical isolation

The preliminary phytochemical tests of the crude methanolic extract were done by the method of Pollock and Stevens [24]. The concentrated crude methanolic extracts (40 g) were partitioned between *n*-butanol and water, while the aqueous part was lyophilized to dryness (≈32 g) and the solvent part was removed under reduced pressure in a rotary evaporator at 45°C. The *n*-butanol fraction, weighing ≈ 35 g, was then purified on silica gel (60–120 mesh, SRL) by column chromatography, and eluted with petroleum ether (PE): PE: CHCl_3_ mixture (at different ratio) CHCl_3_, CHCl_3_: MeOH mixture (at different ratios) and MeOH. All the eluted fractions were monitored by thin-layer chromatography (TLC) using pre-coated aluminium plates (E. Merck, Germany). Two major condensed fractions A and B were isolated along with a mixture of minor compounds in TLC. The isolated major compound(s) were then purified by repeated silica gel column chromatography and were eluted by PE: CHCl_3_ (1:1) and CHCl_3_: MeOH (95:5) mixture to get the pure compound. The spectral analysis of isolated compounds from fraction A and B were done by IR (JASCO-FTIR spectrophotometer in potassium bromide discs), Mass (JEOL JMS600 Mass Spectrometer) and NMR (Bruker DPX-300 NMR spectrometer in DMSO-d_6_ solution). The identification was also done by Co-TLC, and superimposable IR with authentic samples. Melting points were checked in a melting point apparatus by mixed samples, i.e. authentic and isolated ursolic acid and β-sitosterol [20,23].

### Viruses and the cell line

African green monkey kidney cells (Vero cells, ATCC, Manassas, VA, USA) was grown and maintained in Eagle’s minimum essential medium (EMEM), supplemented with 5–10% fetal bovine serum (FBS) [25]. The standard strain HSV-2 G (ATCC-734) and HSV-1 F (ATCC-733), purchased from the ATCC, were used. After plaque purification, the virus was grown and the virus stocks were stored at −80°C for future use [26], and whenever required the virus stocks were grown on Vero cells to determine the titers and used for further study.

### Cytotoxicity assay

Cell toxicity was monitored by determining the effect of the methanolic crude extract, its bioactive fraction A and isolated ursolic acid on cell morphology [27]. Vero cells was cultured onto 96 well plate at 1.0 x10^5^ cells/ml. Different concentrations of methanolic crude extract/fraction A/isolated ursolic acid and standard drug acyclovir were added to each culture wells at a final volume of 100 μl, in triplicate, using DMSO (0.1%) as a negative control. After incubation at 37°C with 5% CO_2_ for 2 days, MTT reagent (10 μl) was added to each well. After 4 h of incubation at 37°C, the formazan was solubilized by adding diluted HCl (0.04 N) in isopropanol, and the absorbance was read at 570 *nm* with a reference wavelength of 690 *nm* by an ELISA reader. Data were calculated as the percentage of cell viability using the formula: [(sample absorbance - cell free sample blank)/mean media control absorbance)]/100%. The 50% cytotoxic concentration (CC_50_) causing visible morphological changes in 50% of Vero cells with respect to cell control were determined [26,28].

### Antiviral assay

The antiviral activity of crude methanolic extract, fraction A and the isolated ursolic acid against HSV-1 and HSV-2 was evaluated by MTT assay [29]. Vero cells were seeded onto 96 well plates with a concentration of 1.0 x10^5^cells/ml. After incubation at 37°C in 5% CO_2_ for 6 h, the virus (0.5 MOI) was added and incubated for 1 h. Different concentrations of crude methanolic extract/fraction A/isolated ursolic acid were added to culture wells in triplicate at a final volume of 100 μl in each well. The maximum concentration of DMSO (0.1%) was used as negative control and acyclovir as positive control throughout the study. After 3 days incubation at 37°C in 5% CO_2_, the MTT test was carried out as described above. Viral inhibition rate was calculated as: [(A_tv_-A_cv_)/(A_cd_-A_cv_)]/100%. A_tv_ indicates the absorbance of the crude methanolic extract/fraction A/ursolic acid with virus-infected cells. A_cv_ and A_cd_ indicate the absorbance of the virus control and the absorbance of the cell control. The antiviral concentration of 50% effectiveness (EC_50_) was defined as the concentration which achieved 50% inhibition of virus-induced cytopathic effects. The amount of virus used in each experiment was based on infected target cells of 0.5 MOI for both the viruses to produce 50% MTT formazan products as in uninfected control cells [30].

### Dose–response assay

To analyze the dose-dependent effect of the test drugs on infected Vero cells, different concentrations of fraction A or isolated ursolic acid was added to HSV-1 and HSV-2 infected Vero cell culture in triplicate. After 2–3 days MTT assay was carried out to determine the inhibition of infection caused by the HSV, as described previously [30,31].

### Viral plaque assay

Plaque reduction assay was used to evaluate the antiviral activity of fraction A or isolated ursolic acid and to compare its activity with acyclovir. This assay evaluated the efficacy of the test agent on inhibition of infection of Vero cells by the free virus particles and thereby the number of viral plaques formed in cell monolayer, as every viral particle non-neutralized by the test agent will infect the cells and formed a plaque. Serial dilutions of fraction A or isolated ursolic acid in EMEM was added to the infected cells (MOI: 0.5 of HSV-1 or HSV-2) and incubated at room temperature, prior to the addition to cells. After 1–2 h incubation at 37°C, the cells were washed with fresh EMEM and overlaid with methylcellulose, so the virus can spread via cell-to-cell route to form plaques. The plaques that developed after 2–3 days of incubation were stained with crystal violet. The effective concentration of fraction A/isolated ursolic acid that inhibited the number of viral plaques by 50% (EC_50_) was interpolated from the dose–response curves [30,31].

### Time response assay

Time response assay was used to investigate the mechanism of inhibition of the infection of HSV by fraction A/isolated ursolic acid at various time periods up to 24 h. Vero cells at 1.0 x10^5^cells/ml were grown onto 96 well plates at 37°C in 5% CO_2_. Following three different approaches the virus (0.5 MOI) was exposed to the different concentrations of the fraction A or isolated ursolic acid before infecting the vero cell (pre-infection); during infection of Vero cell (co-infection); and after the Vero cell culture was infected with the virus (post-infection) in different time interval, in triplicate, using DMSO (0.1%) and acyclovir as a negative and positive control respectively. After incubation at 37°C in 5% CO_2_ for 2–3 days, the MTT test was carried out as described previously [31].

### Immunofluorescence (IFA) study of fraction A treated HSV infected cells

HSV-infected Vero cells monolayer treated with different concentrations of fraction A was washed twice with phosphate buffered saline (PBS, pH 7.2) to remove the cell debris. The cells were then fixed with para-formaldehyde (4%) and blocked with 1% bovine serum albumin (BSA) in 0.1% PBS-triton X100 solution. The cells were washed with PBS, and then permeabilized with 0.1% triton X100 in PBS, and incubated overnight with FITC-labelled anti-HSV-1 mouse monoclonal antibodies (Dako Cytomation, Denmark). After washing with PBS, secondary rabbit polyclonal antibodies (Dako Cytomation, Denmark) and DAPI were added, and the cells were observed under epifluorescence microscope [32].

### Amplification of viral DNA isolated from the infected cells treated with fraction A/isolated ursolic acid by PCR

HSV-1 infected Vero cell cultures, treated with fraction A/isolated ursolic acid at various time intervals (0, 48, 72 h) were harvested. Viral DNA extracted from the tissue culture fluid using QIAmp MiniElute Virus Spin Kit (Qiagen GmbH, Hilden, Germany), was subjected to PCR using HSV-1 type specific primers [33].

### Drug- plant extracts interaction

The antiviral activity of fraction A or isolated ursolic acid, in combination with acyclovir, was evaluated against HSV-1 and HSV-2 (MOI 0.5) by MTT assay, with an aim to know whether this combination can increase the antiviral efficacy. The combined effect of acyclovir and fraction A or isolated ursolic acid on HSV-1 replication was analyzed by isobologram method [34-36]. Here, the EC_50_ was used to calculate the fractional inhibitory concentration (FIC) of the agents in combination. The interaction between fraction A or isolated ursolic acid and acyclovir was interpreted according to the combined FIC index [FIC_extract/compound_ + FIC_acyclovir_ as synergy (≤0.5), no interaction (0.5-4) or antagonism (>4) [37].

### Statistical analysis

The selective index (SI), a marker of antiviral activity, was determined as the ratio of CC_50_ to EC_50_. The statistically different effects of crude methanolic extract/fraction A or isolated ursolic acid and acyclovir on the inhibition of HSV were compared with the control group as well as between fraction A or isolated ursolic acid, using Student’s *t*-test. While the dose-dependent effect of antiviral activity was determined by linear regression.

## Results

### Spectral analysis of isolated compounds from fraction A and fraction B

**Figure 1:**
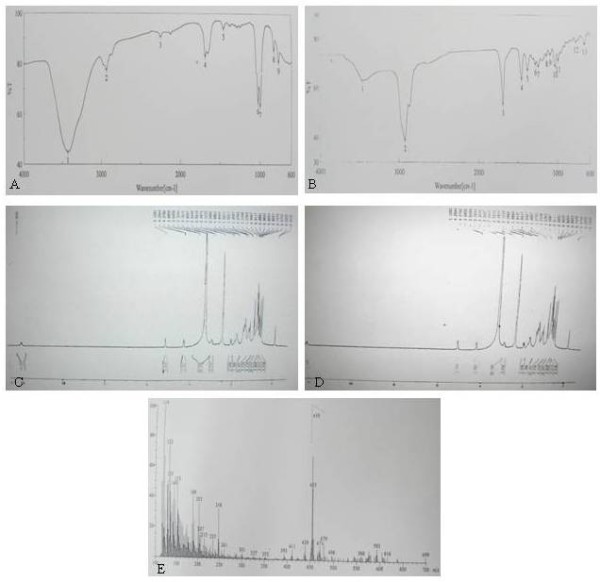
**IR,**^**1**^**HNMR and Mass Spectra of isolated compound from fraction A with authentic ursolic acid.** The concentrated crude methanolic extract was partitioned between *n*-butanol and water. The aqueous part was lyophilized while the solvent part was evaporated by Eyela (Uni trap UT 1000, Japan) rotary evaporator at 45°C. The *n*-butanol fraction was purified on silica gel column, and eluted with PE: PE: CHCl_3_, CHCl_3_: MeOH and MeOH. The eluted fractions, monitored by TLC, yielded two major condensed fractions **A** and **B**. The isolated compound(s) from fraction **A** was then purified by repeated silica gel column and eluted with PE: CHCl_3_ (1:1) and CHCl_3_: MeOH (95:5) mixture to obtain pure compound. The IR spectra of isolated compounds from fraction **A** done by JASCO-FTIR spectrophotometer in potassium bromide discs [A] agreed well with the authentic sample of ursolic acid [**B**]. The ^1^HNMR spectrum of isolated compound from fraction **A** by Bruker DPX-300 NMR spectrometer in DMSO-d_6_ solution [**C**] indicated that the isolated compound was almost identical with the authentic sample of ursolic acid [**D**]. The Mass spectra of isolated compound from fraction A, determined by JEOL JMS600 Mass Spectrometer [**E**] indicated the compound to be ursolic acid.

The spectral data (IR, Mass and NMR) and melting points of isolated compounds from fraction A and B were identical with ursolic acid and β-sitosterol, respectively. The *IR spectra* of the isolated compound from fraction A (Figure 1A) agreed well with the authentic sample of ursolic acid (Figure 1B). The spectrum showed absorption band at 3458 and 1696 cm^-1^ indicating the presence of hydroxyl and carboxyl groups; while the band at 1033 and 996 cm-^1^ indicated –C-OH bond, and another peak at 2929 cm^-1^ arises from the C-H bonds.

The ^*1*^*HNMR spectrum* of the isolated compound from fraction A in DMSO-d_6_, showed the signal at 11.92 indicating a –COOH group at 28^th^ position. While the signal at δ3.3 agrees with the presence of –CH-OH at 3^rd^ position, and –OH at δ4.29 peak. Signal at δ5.12 signifies the presence of a trisubstituted double bond (unsaturation) and the seven –CH_3_ groups between δ0.6 and 1.3. The shift positions of the isolated compound were almost identical with the authentic sample of ursolic acid (Figure 1C, 1D). The *mass spectra* of the compound isolated from fraction A showed prominent peak at *mlz* 248, and other peaks at *mlz* 207, 203 and 189 indicated the compound to be ursolic acid (Figure 1E). The *melting point* of isolated compound is 285–287°C, similar to the authentic sample of ursolic acid.

**Figure 2:**
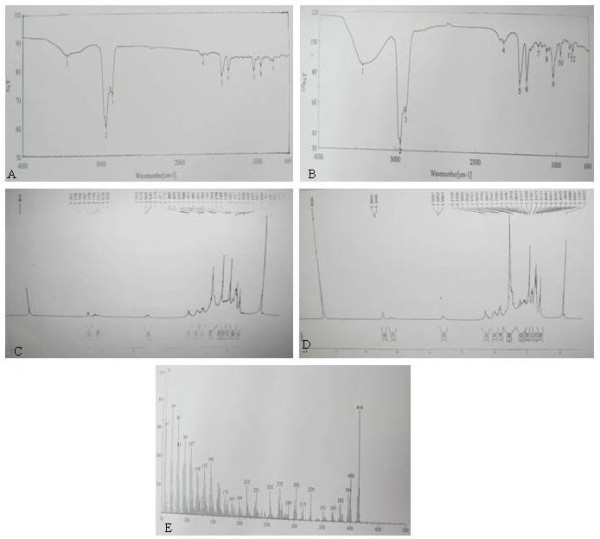
**IR,**^**1**^**HNMR and Mass Spectra of isolated compound from fraction B with authentic β-sitosterol.** The concentrated crude methanolic extract was partitioned between *n*-butanol and water. The aqueous part was lyophilized while the solvent part was evaporated by Eyela (Uni trap UT 1000, Japan) rotary evaporator at 45°C. The *n*-butanol fraction was purified on silica gel column, and eluted with PE: PE: CHCl_3_, CHCl_3_: MeOH and MeOH. The eluted fractions, monitored by TLC, yielded two major condensed fractions **A** and **B**. The isolated compound(s) from fraction **B** was then purified by repeated silica gel column and eluted with PE: CHCl_3_ (1:1) and CHCl_3_: MeOH (95:5) mixture to obtain pure compound. The IR spectra isolated compounds from fraction B done by JASCO-FTIR spectrophotometer in potassium bromide discs [**A**] agreed well with the authentic sample of β-sitosterol [**B**]. The ^1^HNMR spectrum of isolated compound from fraction B by Bruker DPX-300 NMR spectrometer in DMSO-d_6_ solution [**C**] indicated that the isolated compound was almost identical with the authentic sample of β-sitosterol [**D**]. The Mass spectra of isolated compound from fraction **B**, determined by JEOL JMS600 Mass Spectrometer [**E**] indicated the compound is β-sitosterol.

The *IR spectrum* of the isolated compound from fraction B shows absorption bands at 3426 and 1056 cm^-1^ indicating the presence of –OH group. Other prominent peaks were at 2935, 2852, 1706 and 1462 cm^-1^ arising from the hydrocarbon skeleton. The absorption band at 965 and 802 cm^-1^ is due to C = C-H group (Figure 2A, 2B). The ^*1*^*HNMR spectrum* of the isolated compound from fraction B shows the shift at δ3.53, indicating CH-OH group at C3 position. The olefinic proton at 6^th^ position has peak at δ5.34 and the six –CH_3_ group appeared between δ0.6–1.03 regions. Other protons appeared between δ1.0 - 2.3. The shift position indicated that the isolated compound was almost identical with the authentic sample of β-sitosterol (Figure 2C, 2D). The *mass spectra* of isolated compound had the peak at *mlz* 414 (M+) with significant fragment ion peaks at *mlz* 396, 382, 273 255, 231, and 213. The intense peak with highest mass number at *mlz* 414 is due to parent molecular ion β-sitosterol (Figure 2E). A less intense peak at *mlz* 400 signifies the presence of its lower homologue (campesterol) in small amount. The *melting point* for isolated compound was 136–137°C, similar to β-sitosterol authentic sample.

### Assessment of cytotoxicity and anti-HSV activity by MTT assay on Vero cell

The MTT assay was used to determine the toxicity of the tested agents. The results revealed that the crude methanolic extract of *M. peltatus*, its fraction A and isolated ursolic acid exhibited a cytotoxic effect on Vero cells at concentrations higher than their EC_50_. Results presented in Table 1 revealed that the CC_50_ of crude methanolic extract, fraction A and isolated ursolic acid were 452 μg/ml, 174 μg/ml and 110 μg/ml respectively. The anti-HSV activity tested by MTT assay showed that the crude methanolic extract, fraction A and the isolated ursolic acid had anti-HSV activity at different dose level, based on their EC_50_ value and selectivity index (SI). The EC_50_ of fraction A (7.8 ± 1.6 and 8.2 ± 1.8), and isolated ursolic acid (5.5 ± 0.54 and 5.8 ± 1.1) against HSV-1 and HSV-2 revealed the strongest anti-HSV activity, compared to the crude methanolic extract (p < 0.0001). Further, the EC_50_ and SI index indicated that fraction A and isolated ursolic acid was more active against HSV-1 than HSV-2. On the otherhand, fraction B had CC_50_ and EC_50_ at higher concentration with very low SI index, indicating its inactiveness, compared to acyclovir (Table 1).

**Table 1:** **Assessment of Anti-HSV activity of*M. peltata*crude methanolic extract and its constituents by MTT assay**

**Test drug**	**CC_50_^a^**	**HSV-1 F at MOI: 0.5**		**HSV-2 G at MOI: 0.5**	
		**Antiviral activity (EC_50_^b^)**	**Selectivity index (SI)^c^**	**Antiviral activity (EC_50_^b^)**	**Selectivity index (SI)^c^**
Crude methanolic extract	452	57.5 ± 2.45	7.86	61.2 ± 3.1	7.38
Fraction A	174	7.8 ± 1.6	22.3	8.2 ± 1.8	21.21
Fraction B	215	105.0 ± 5.7	2.04	120.0 ± 7.2	1.79
Ursolic acid (isolated)	110	5.5 ± 0.54	20	5.8 ± 1.1	18.97
Acyclovir	130	2.1 ± 0.1	61.9	2.9 ± 0.1	44.8

^a^ The 50% cytotoxic concentration for Vero cells in μg/ml.

^b^ Concentration of compound (μg/ml) producing 50% inhibition of virus induced CPE of three separate experiments.

^c^ Selectivity index (SI) = CC_50_/EC_50_.

### Dose-effect of fraction A/ursolic acid

**Figure 3:**
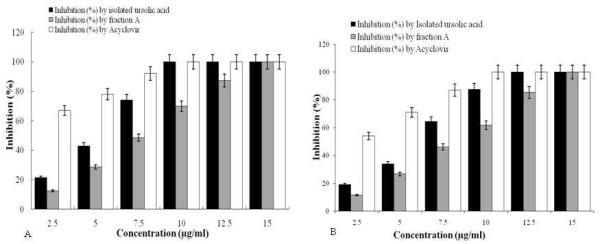
**Dose dependent activity of isolated ursolic acid or fraction-A and acyclovir on HSV-1 [A] and HSV-2 [B].** Different concentrations of fraction **A**, isolated ursolic acid or acyclovir (2.5-15 μg/ml) were added to HSV-1 and HSV-2 (black, grey and white bars) infected Vero cells after 1 h of infection. Inhibition percentage was evaluated by MTT assay after 3 days of incubation at 37°C. Each bar represents the mean ± S.E.M of three independent experiments.

To analyze the dose-dependent antiviral activity we used different concentrations of fraction A, isolated ursolic acid, along with acyclovir and DMSO (0.1%) as positive and negative control respectively, on HSV-1 and HSV-2 infected Vero cells. The results presented in Figure 3A, showed that fraction A at 14.5 μg/ml and isolated ursolic acid at 9.0 μg/ml exhibited nearly 100% inhibition against HSV-1. Similar effect was noticed a gap between at and 15 isolated ursolic acid at 12.5 μg/ml against HSV-2 (Figure 3B), indicating a high correlation between drug concentration and inhibition rate.

### Assessment of plaque reduction assay

**Figure 4:**
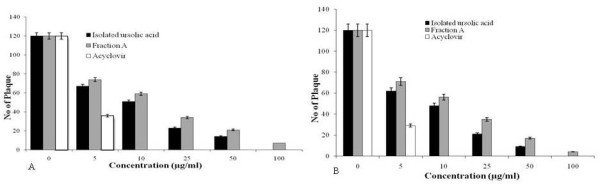
**Plaque reduction assay of HSV-1[A] and HSV-2[B] with fraction A, isolated ursolic acid and acyclovir.** Different concentrations of fraction **A**, isolated ursolic acid or acyclovir (5–100 μg/ml) were added to HSV-1 and HSV-2 (black, grey and white bars) infected Vero cells. After 1-2 h incubation at 37°C the cells were overlaid with methylcellulose and the plaques developed after 2–3 days of incubation were stained with crystal violet. The plaque number inhibition was calculated, and the effective concentration of fraction A/isolated ursolic acid that inhibited the number of viral plaques was interpolated from the dose–response curve.

Plaque reduction assay was also used to access the antiviral activity of fraction A, and isolated ursolic acid, using acyclovir and DMSO (0.1%) as positive and negative control respectively. The results revealed that both fraction A and isolated ursolic acid at a concentrations of 5–100 μg/ml inhibited plaque formation by HSV-1 and HSV-2, indicating their dose dependent inhibitory activity (Figure 4). The control drug acyclovir showed 100% inhibition of plaque formation at 10 μg/ml, while no inhibition was noticed with 0.1% DMSO (data not shown).

### Time course analysis of fraction A and or isolated ursolic acid

**Figure 5:**
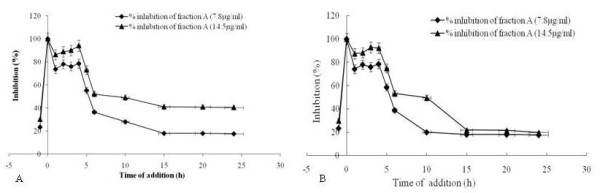
**Inhibitory effect of fraction A during pre-infection, co-infection and post-infection on HSV-1 [A] and HSV-2 [B].** Different concentrations of fraction A [7.8 μg/ml (square), and 14.5 μg/ml (triangle)] were added with the HSV-1 and HSV-2 infected Vero cells at various time period like pre-infection (−1 h), co-infection (0 h) or post-infection (1-24 h). After 3 days of incubation at 37°C, inhibition was evaluated by MTT assay and expressed as the inhibition percentage. Each bar represents the mean ± S.E.M of three independent experiments.

Time course analysis was performed with fraction A or isolated ursolic acid to investigate the mechanism of antiviral activity. Inhibition was evaluated by MTT assay after 3 days of infection and expressed as percentage inhibition. The result showed that fraction A at 7.8 and 14.5 μg/ml inhibited HSV-1 (Figure 5A) and HSV-2 (Figure 5B) significantly (p < 0.001) within 2–5 h post-infection, i.e., during the early period of virus multiplication. Whereas no inhibition was found when the virus was exposed to fraction A or isolated ursolic acid before infection (pre-infection) or together (co-infection), upto the highest concentration tested.

### Immunofluorescence (IFA) study with fraction A treated HSV infected cells

**Figure 6:**
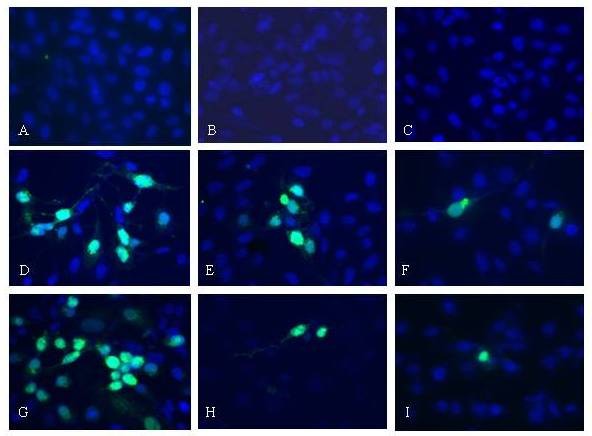
**Immunofluorescence study of HSV-1 infected Vero cells treated with fraction-A for 2–4 h post-infection.** Vero cell Control [**A**]; Cells treated with fraction **A** (14.5 μg/ml) for 2 h [**B**] and 4 h [**C**]; Virus control at 2 h post-infection [**D**]; Virus infected cell treated with fraction A at 7.8 μg/ml [**E**], and 14.5 μg/ml [**F**] at 2 h post-infection; Virus Control at 4 h post-infection [**G**]; Virus infected cell treated with fraction A at 7.8 μg/ml [**H**] and 14.5 μg/ml [**I**] at 4 h post-infection.

Indirect immunofluorescence assay was used to determine the kinetics of most active fraction A on antigen expression of HSV-1 F. The HSV-1 F infected Vero cells were treated with different concentrations of fraction A and incubated for different time intervals. The results revealed less number of virus particle in Vero cells treated with the fraction A, indicating the strong anti-HSV activity. The HSV antigen expression showed a characteristic pattern of small foci of single fluorescent in fraction A treated HSV-1 infected cells at different time interval (2-4 h post-infection), suggesting drug inhibited viral dissemination (Figure 6). Moreover, the antiviral activity of fraction A is more evident at its highest concentration (14.5 μg/ml) tested.

### Amplification of viral DNA isolated from HSV-1 infected Vero cells treated with fraction A or isolated ursolic acid by PCR

**Figure 7:**
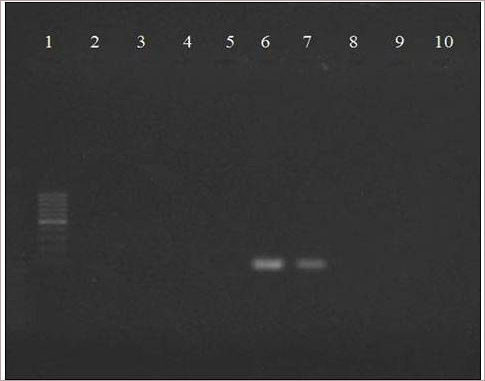
**Detection of HSV-1 DNA in fraction A/ursolic acid or acyclovir-treated and untreated cultures by PCR.** Lane 1: 100 bp Marker; Lane 2: PCR control; Lane 3: cell control; Lane 4: cell + fraction A; Lane 5: cell + acyclovir; Lanes 6: positive control (HSV-1 after 72 h); Lane 7: cell + HSV-1 after 48 h; Lane 8: cell + HSV-1 (MOI: 0.5) + fraction A (14.5 μg/ml); Lane 9: cell + HSV-1 (MOI: 0.5) + isolated ursolic acid (9 μg/ml); Lane 10: cell + HSV-1 (MOI: 0.5) + acyclovir (5 μg/ml).

To compare the effect on viral replication, DNA amplification of HSV-infected and fraction A or isolated ursolic acid and acyclovir treated HSV-1 was detected by PCR. The results demonstrated that the fraction A/isolated ursolic acid treated HSV-1 (MOI 0.5) cultures at 24-72 h duration failed to show any amplification, similar to acyclovir (drug control) treated cultures. While HSV-1 infected culture (control) showed clear amplification of viral DNA in 1% agarose gel at 48 h and 72 h (Figure 7). Furthermore, in infected cells, amplification of *pol* gene (internal control) indicated the integrity of the gene.

### Drug- plant extracts interaction

To evaluate whether the fraction A and or isolated ursolic acid can able to increase the inhibitory efficacy of acyclovir in combination, we have tested the fraction A-acyclovir and isolated ursolic acid-acyclovir combination (synergism) by MTT assay, using isobologram method. Our results demonstrated that the EC_50_ of fraction A, isolated ursolic acid and acyclovir was 7.8, 55 and 2.1 μg/ml respectively, but in combination with acyclovir the mean EC_50_ was 2.7 and 2.51 μg/ml respectively. Moreover, the FIC index of 0.78 (between acyclovir and fraction A) and 0.84 (between acyclovir and isolated ursolic acid), indicated that there was no synergistic interaction between them (Table 2). Furthermore, none of these combinations exhibited cytotoxic effect against Vero cell (data not shown).

**Table 2:** Effects of fraction A/isolated ursolic acid in combination with acyclovir on HSV-1 F infected Vero cells

**Fraction/Compound**	**Mean EC_50_ ± SD^a^**	**FIC_fraction/compound_ + FIC_acyclovir_^b^**	**Inhibitory effect**
Fraction A	7.8 ± 1.6	-	-
Ursolic acid (isolated)	5.5 ± 0.54	-	-
Acyclovir alone	2.1 ± 0.1	-	
Acyclovir + Fraction A	2.7 ± 0.15	0.84	No interaction
Acyclovir + isolated ursolate	2.51 ± 0.12	0.78	No interaction

^a^ Results are the mean of three independent experiments of MTT assay.

^b^ FIC_fraction/compound_ + FIC_acyclovir_ are FICs of fraction/compound and acyclovir, respectively.

## Discussion

The present study for the first time, demonstrated the anti-HSV activity of crude methanolic extract of *M. peltatus* leaf, an ethnomedicine of Onge tribes of Andaman and Nicobar Islands, India. Phytochemical study revealed that the crude methanolic extract contain two major fractions, fraction A and B, of which fraction A had significant anti-HSV activity. Chromatographic separation and spectral analysis revealed that fraction A contains a known triterpene ursolic acid, which possesses strong antiviral activity against HSV-1 and HSV-2 *in vitro*. The antiviral activity of the crude methanolic extract was weak compared to fraction A, probably due to its low concentrations of bioactive compound(s). While the higher antiviral activity of fraction A, than the crude methanolic extract, is due to the higher concentration of bioactive compounds within the fraction. Earlier study with the crude methanolic extract of *M*. *peltatus* showed moderate antibacterial and antifungal [20], analgesic and antiinflammatory [20,21,38] activity. On the otherhand fraction B do not showed any anti-HSV activity, hence not included in further study.

The cytotoxicity study revealed that the crude methanolic extract, fraction A and isolated ursolic acid had different CC_50_ due to the variable concentration of bioactive compound(s), and the antiviral activity was found far below the CC_50_ dose. Further, fraction A and isolated ursolic acid on both HSV-1 F and HSV-2 G revealed dose-dependent antiviral activity. Infection of Vero cell by HSV-1 and HSV-2 was significantly prevented by the fraction A, or isolated ursolic acid with higher SI values. However, the CC_50_ of fraction B was just double the EC_50_, giving an insignificant SI for both HSV-1 and HSV-2.

The dose-dependent activity and time course analysis was done to understand how the fraction A and isolated ursolic acid inhibit the viral infection. Interestingly, fraction A and isolated ursolic acid was found to inhibit both HSV-1 and HSV-2 infection(s) in dose-dependent manner, with an EC_50_ of 7.8 μg/ml and 5.5 μg/ml for HSV-1 F and 8.2 and 5.8 μg/ml for HSV-2 G, respectively. However, 100% inhibition of HSV-1 was recorded at 14.5 μg/ml of fraction A and 9.0 μg/ml for isolated ursolic acid; while for HSV-2 it was 15 μg/ml of fraction A and 12.5 μg/ml of isolated ursolic acid. Results on the time-course effect between 0 and 5 h post-infection revealed that the fraction A and isolated ursolic acid possess a similar inhibitory effect. This suggests that the mode of action is not due to inhibition of vial adsorption, but inhibition of viral replication. The time response study, also demonstrated that both fraction A and isolated ursolic acid probably interfere early stage of HSV replication, as the maximum inhibition was noted at 2-5 h post-infection. However, the real antiviral mechanism of fraction A and isolated ursolic acid remains to be further elucidated.

The indirect immunofluorescence assay was carried out to determine the kinetics of fraction A on antigen expression of HSV-1. Maximum reduction in number of infected fluorescent cells was observed at 4 h, along with a characteristic pattern of small foci of positive cells and even single fluorescent cells. This suggests that fraction A can inhibit viral dissemination. When fraction A was subsequently added at 2 h and 4 h time period, a significant reduction of positive fluorescent cells was observed (Figure 6). The non-amplification of fraction A treated HSV-1 infected cells by PCR further complemented and strengthened the antiviral activity of this plant. Detection and amplification of *pol* gene (control gene) in fraction A or isolated ursolic acid treated and virus infected cells (Figure 7) indicated that there was no cytotoxicity after treatment of cells with this plant product.

The widely used anti-herpes virus drug acyclovir is a nucleoside analogue, specifically targets the thymidine kinase of HSV [39]. However, its extensive and long term use yielded drug-resistant strains [9,11], due to mutations in viral *thymidine kinase* and/or *DNA polymerase*, that alter substrate sensitivity [40], and thus, become chromosome mutagen. Moreover, efficacy of therapeutic vaccines against primary and recurrent HSV infection has failed [15] and thus, search for natural alternative is the top priority to control and prevent HSV infections and its transmission. The earlier reports indicated that different species of *Mallotus* (*M. philippinensisM. japonicusM. repandus*) contain several secondary metabolites like terpenoids (mallotucin and malloripine), diterpenic lactones (mallotucin B,C,D), triterpene alcohol (moretenol), saponins (crotoxigenin, coroglusagenine), cardinolipids, resins (rottlerine, isorottlerine), flavonoids, and β-sitosterol [41,42]. However, there were no reports on the bioactivity and phytochemistry of *M. peltatus*, except the isolation of ursolic acid and β-sitosterol alongwith some fatty acids by this group [20]. The ursolic acid is a pentacyclic amphiphilic triterpene with planner hydroxylated polycyclic [(3b)-3-hydroxyurs-12-en-28-oic acid] structure, ubiquitous in medicinal plants as free acid or aglycones for triterpenoid saponins, and have been used since antiquity due to multiple bioactivities [21]. Contemporary research revealed that the ursolic acid, isolated from plants, is cytotoxic to some tumor and cancer cells [43-45], including skin tumor [46] and recommended for skin cancer therapy [47]. Other studies reported its antiviral [48], antibacterial [49], and potent anti-inflammatory [50-52] activities. It is a highly selective inhibitor of cyclic AMP-dependent protein kinase [53], human DNA polymerases and DNA topoisomerases [54] and has antioxidative [55] and apoptotic [56,57] activities. Ursolic acid isolated from *Rosmarinus officinalis* L. leaves is reported to inhibit the motility of *Trypanosoma cruzi* epimastigotes [58]; while ursolic acid isolated from *Ocimum sanctumO. basilicum* and *O. americanum* showed anti-HSV activity with ED_50_ of 35–47 μg/ml by interfering at various steps of viral multiplication [59].

Thus, our results showed that fraction A, which contain ursolic acid as one of the compound, might be a potential therapeutic candidate against HSV infections, as indicated by its SI value (7.86 – 22.3). Ursolic acid is known to be less toxic, can restore skin’s collagen bundle and elasticity, and is dermatologically innocuous [60], while the antivirals presently used for herpes virus treatment have high toxicity, several side effects and problem of frequent drug resistance development. Therefore, our study demonstrated that the fraction A and its component(s) can serve as an alternative agent in herpes virus infection and thus, merit a greater attention.

## Abbreviations

HSV, Herpes simplex virus; DMSO, Dimethyl sulfoxide; MOI, Multiplicity of infection; MTT, (3-(4,5-Dimethylthiazol-2-yl)-2,5-diphenyltetrazolium bromide; HCl, Hydrochloric acid; TCID, Tissue culture infective dose; CC50, Cytotoxic concentration that is toxic to 50% cells; EC50, Effective concentration required to achieve 50% protection against virus-induced cytopathic effect; SI, Selectivity index; FIC, Fractional inhibitory concentration; CPE, Cytopathic effect.

## Competing interests

The authors declare that they have no competing interests.

## Authors’ contributions

PB, HM, DO, and NM contributed in lab work and DC in manuscript write up. MCS, and SC provided facility for some lab work, while TKC, GD help in some data analysis and technical details. DC was the principal investigator who planned and monitored the work and provided all the facilities to complete this work. All the authors read and approved the final manuscript.

## Authors’ information

Paromita Bag, Durbadal Ojha (M. Sc Microbiology), Nilanjan Mandal (M. Sc Biochemistry), Hemanta Mukherjee (M. Pharm, Clinical Pharmacy), Mamta Chawla Sarkar (PhD Zoology), Sekhar Chakraborti (PhD Biochemistry), Tapan Chatterjee (PhD Pharmacy), Gobardhan Das (PhD Immunology) and Debprasad Chattopadhyay (PhD Pharmaceutical Microbiology, and Assistant Director of ICMR Virus Unit, Kolkata).
